# A Novel Pyroptosis-Related Signature for Predicting Prognosis and Indicating Immune Microenvironment Features in Osteosarcoma

**DOI:** 10.3389/fgene.2021.780780

**Published:** 2021-11-26

**Authors:** Yiming Zhang, Rong He, Xuan Lei, Lianghao Mao, Pan Jiang, Chenlie Ni, Zhengyu Yin, Xinyu Zhong, Chen Chen, Qiping Zheng, Dapeng Li

**Affiliations:** ^1^ Department of Orthopedics, Affiliated Hospital of Jiangsu University, Zhenjiang, China; ^2^ Cancer Institute, The Affiliated People’s Hospital of Jiangsu University, Zhenjiang, China; ^3^ Department of Burn and Plastic Surgery, Affiliated Hospital of Jiangsu University, Zhenjiang, China; ^4^ Guizhou Orthopedics Hospital, Guiyang, China; ^5^ Department of Hematological Laboratory Science, Jiangsu Key Laboratory of Medical Science and Laboratory Medicine, School of Medicine, Jiangsu University Zhenjiang, Guiyang, China; ^6^ Shenzhen Academy of Peptide Targeting Technology at Pingshan, and Shenzhen Tyercan Bio-Pharm Co., Ltd., Shenzhen, China

**Keywords:** osteosarcoma, pyroptosis, prognosis, immunotherapy, survival analysis

## Abstract

Osteosarcoma is a common malignant bone tumor with a propensity for drug resistance, recurrence, and metastasis. A growing number of studies have elucidated the dual role of pyroptosis in the development of cancer, which is a gasdermin-regulated novel inflammatory programmed cell death. However, the interaction between pyroptosis and the overall survival (OS) of osteosarcoma patients is poorly understood. This study aimed to construct a prognostic model based on pyroptosis-related genes to provide new insights into the prognosis of osteosarcoma patients. We identified 46 differentially expressed pyroptosis-associated genes between osteosarcoma tissues and normal control tissues. A total of six risk genes affecting the prognosis of osteosarcoma patients were screened to form a pyroptosis-related signature by univariate and LASSO regression analysis and verified using GSE21257 as a validation cohort. Combined with other clinical characteristics, including age, gender, and metastatic status, we found that the pyroptosis-related signature score, which we named “PRS-score,” was an independent prognostic factor for patients with osteosarcoma and that a low PRS-score indicated better OS and a lower risk of metastasis. The result of ssGSEA and ESTIMATE algorithms showed that a lower PRS-score indicated higher immune scores, higher levels of tumor infiltration by immune cells, more active immune function, and lower tumor purity. In summary, we developed and validated a pyroptosis-related signature for predicting the prognosis of osteosarcoma, which may contribute to early diagnosis and immunotherapy of osteosarcoma.

## Introduction

Osteosarcoma is the most common primary aggressive malignancies of the skeleton, and it occurs mainly in children and adolescents, in which distant metastasis still leads to a poor prognosis (Chow et al., 2020; Rojas et al., 2021). With a combination of neoadjuvant chemotherapy, surgery, chemotherapy, and biological therapy in the last few years, the 5-year survival rate for osteosarcoma patients has improved significantly, from 20% to 65–70% (Yao and Chen, 2020; Gazouli et al., 2021). However, due to the limited efficacy of current treatment strategies, nearly 30% of osteosarcoma patients are prone to metastasis or recurrence, with poor prognosis and low 5-year survival rates (Fan et al., 2021). Recently, immunotherapy has undergone a dramatic transformation, demonstrating superior anticancer efficacy in many tumors and being recognized as a more potent and antigen-specific form of antitumor therapy (Constantinidou et al., 2019; Shin et al., 2021). For example, adoptive cellular immunotherapy is a promising option for tumors resistant to current conventional therapy, and chimeric antigen receptor T-cell therapy has been shown to cure 25–50% of patients with previously incurable B-cell malignancies, revolutionizing the treatment of drug-resistant hematologic malignancies (Titov et al., 2021). In addition, specific immune checkpoint inhibitors are being explored as new immunotherapeutic strategies for osteosarcoma, such as CTLA-4, LAG3, TIGIT, and PD-1/L1 (Wang S.-D. et al., 2016; Hashimoto et al., 2020; Judge et al., 2020; Park and Cheung, 2020; Ligon et al., 2021). However, cancer immunotherapies, including checkpoint inhibitors, have varying response rates due to multiple primary and acquired resistance mechanisms (Bashash et al., 2021). In order to improve the early diagnosis and treatment of osteosarcoma, novel biomarkers and therapeutic targets are needed.

Pyroptosis is a newly discovered form of programmed cell death that is morphologically distinct from apoptosis and necrosis while releasing inflammatory mediators in the process (Wu et al., 2021). Pyroptosis is mediated by pore-forming proteins, such as the gasdermin family, of which gasdermin D (GSDMD) is a primary substrate for the caspase family (Li L. et al., 2021). After cleavage by activated caspases, the N-terminal fragment of GSDMD oligomerizes in the membrane to form pores, leading to pyroptosis (Lu et al., 2021). Pyroptosis acts as a double-edged sword in cancer. On the one hand, pyroptosis can create a tumor-promoting environment by releasing inflammatory factors; on the other hand, pyroptosis can inhibit tumor occurrence and progression as a form of programmed death (Xia et al., 2019). As research progresses, the impact of pyroptosis-related genes on the proliferation, migration, and invasion of tumor cells becomes increasingly prominent and is strongly associated with cancer prognosis (Ju A. et al., 2021; Lin W. et al., 2021; Shao et al., 2021; Ye et al., 2021). For instance, Tang et al. (2020) reported that pyroptosis inhibited metastasis of colorectal cancer cells through activation of NLRP3-ASC-Caspase-1 signaling by FL118. In another study by Wang Y. et al. (2016), it was found that the NLRP3 inflammasome can promote the proliferation and migration of A549 lung cancer cells via the caspase-1-IL-1β/IL-18 signaling pathway. Studies have shown that GSDMD was notably upregulated in osteosarcoma compared to normal skeletal tissue as well as associated with drug resistance and prognosis for patients with osteosarcoma (Lin R. et al., 2020). Alternatively, GSDMD expression was significantly downregulated in gastric cancer tissues, which may contribute to the development of gastric cancer through the regulation of cell cycle transition (Wang et al., 2018). However, the mechanism of pyroptosis-related genes in osteosarcoma is still not fully elucidated.

Recently, high-throughput sequencing technologies and bioinformatics analysis have enabled the exploration of genetic alterations in osteosarcoma and provided an effective way to identify potentially beneficial markers and the most appropriate treatment strategies for other cancer types (Li M. et al., 2021; Na et al., 2021; Pan et al., 2021). According to Zhang et al. (Xing et al., 2021), TIMELESS was the most significantly upregulated gene within the 16 clock-related genes by analyzing The Cancer Genome Atlas (TCGA) database and promoted cancer cell proliferation and migration via increasing macrophage infiltration in ovarian cancer. An analysis of the relationship between osteosarcoma development and KIF21B using bioinformatics analysis showed that knockdown of KIF21B inhibited cell proliferation and reduced tumor formation *in vivo* by modulating the PI3K/AKT pathway and that KIF21B was an independent prognostic factor in osteosarcoma patients (Ni et al., 2020). The previous success of projects to identify prognostic target genes suggests that it may be possible to uncover more molecular mechanisms in osteosarcoma.

We used microarray data from the Therapeutically Applicable Research to Generate Effective Treatments (TARGET) and Genotype-Tissue Expression (GTEx) database for differential expression analysis and identified 46 differentially expressed pyroptosis-related genes (DEPRGs) in osteosarcoma and normal muscle tissues. We then constructed a six-gene signature (that could determine the PRS-score) based on DEPRGs to predict osteosarcoma outcomes. We validated the signature by evaluating the association between the PRS-scores and clinical characteristics and immune microenvironment features in osteosarcoma tumors. The differential genes among the PRS-score-based subgroups are also enriched for immunological functions and may be involved in regulating the composition of the immune microenvironment. These results reveal that the pyroptosis-related prognostic signature may provide new insights into osteosarcoma diagnosis and prognosis prediction.

## Materials and Methods

### Data Acquisition

**FIGURE 1:**
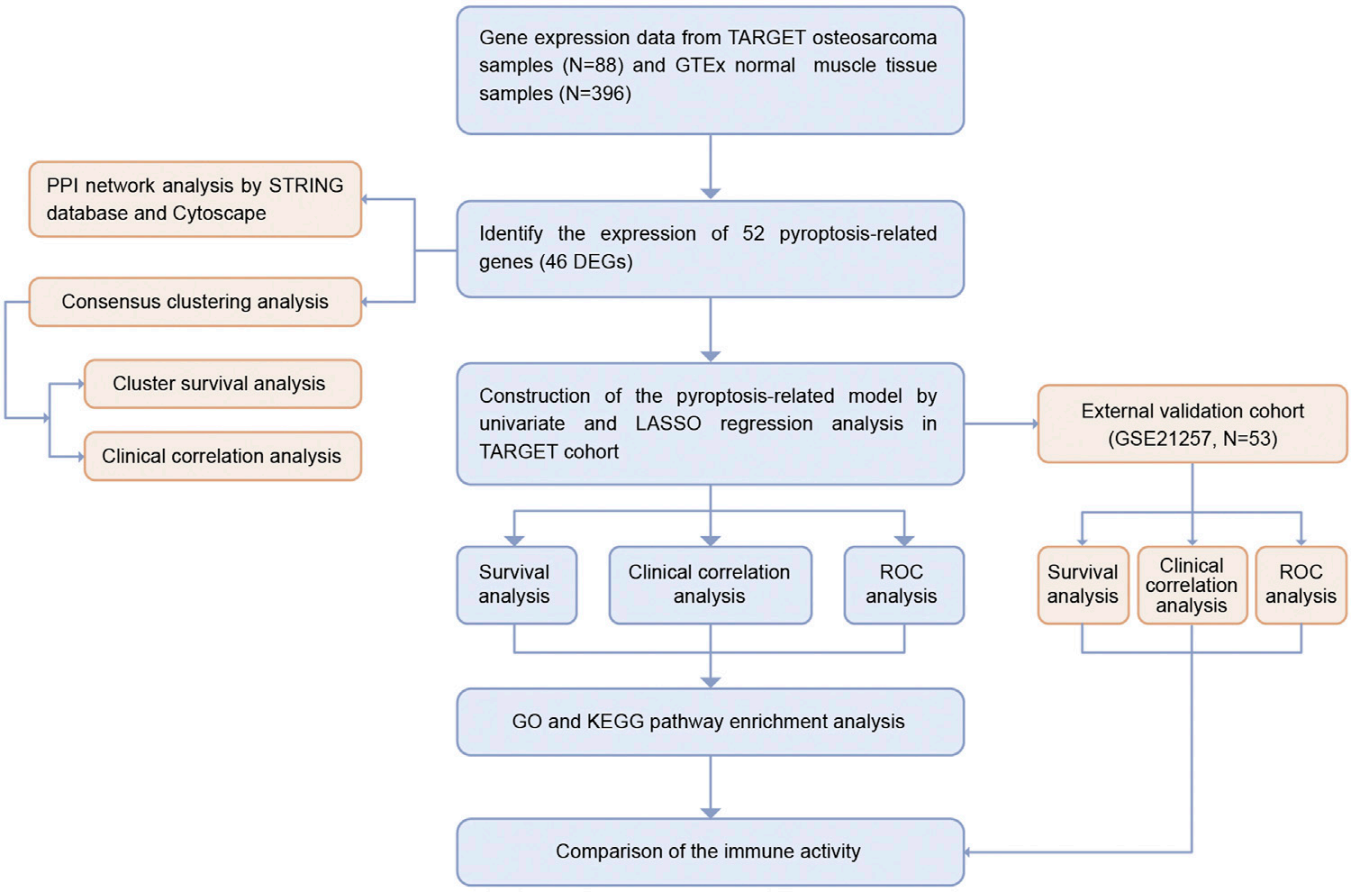
Flow chart of the study.

The workflow chart of this study is shown in Figure 1. We extracted the RNA sequencing (RNA-seq) data and the corresponding clinical information of 88 osteosarcoma patients from the TARGET database (https://ocg.cancer.gov/programs/target). The RNA-seq data of 396 normal human muscle tissue samples were obtained from the GTEx database (https://xenabrowser.net/datapages/). Both data types were HTseq-FPKM, and all gene expression levels were processed with log^2^ (FPKM + 1). The independent cohort GSE21257, which contained 53 osteosarcoma samples, was downloaded from Gene Expression Omnibus (GEO) database (https://www.ncbi.nlm.nih.gov/geo/query/acc.cgi?acc=GSE21257).

### Identification of DEPRGs

We obtained 52 pyroptosis-related genes (PRGs) from prior reviews (Xia et al., 2019; Zhou and Fang, 2019; Li et al., 2020; Ju X. et al., 2021; Wu et al., 2021; Ye et al., 2021) and MSigDB database v7.4 (Subramanian et al., 2005) (listed in Supplementary Table S1). We identified DEPRGs between tumor and normal tissues using the “limma” package, with a *p*-value < 0.05. A protein-protein interaction (PPI) network of all DEPRGs was obtained by STRING database (http://www.string-db.org/). We used Molecular Complex Detection (MCODE), a plugin for Cytoscape, to cluster the genes and find a densely connected area based on the following criteria: degree cut-off = 2, haircut on, node score cut-off = 0.2, Max depth = 100, k-score = 2, score ≥ 5, and node ≥ 10.

### Consensus Clustering Analysis

We downloaded all clinical data from the TARGET dataset and further analyzed a total of 85 patients with survival time and status. We performed consensus clustering analysis based on the clinical characteristics of osteosarcoma patients in the TARGET dataset using the “ConsensusClusterPlus” package. The clustering index “k” was increased from 2 to 10 to identify the clustering index with the minor interference and the greatest difference between clusters.

### Construction of a Pyroptosis-Related Scoring Signature

We conducted univariate Cox analysis with the “survival” package to screen for prognosis-related DEPRGs and set 0.1 as the threshold *p*-value for omission prevention (Ye et al., 2021). We then conducted the LASSO Cox regression analysis to narrow the risk of overfitting to develop a prognostic signature using “glmnet” package. The TARGET osteosarcoma patients were divided into low and high PRS-score groups based on the median PRS-score, and the PRS-score formula was as follows: PRS-score = *Σ* (βi × Expi) (β: coefficients, Exp: gene expression level). We created a Kaplan–Meier survival curve using the R “survival” and “survminer” packages to determine the OS time between the two subgroups. The principal component analysis (PCA) based on the signature was performed using the R package “Rtsne” and “ggplot2”. The specificity and sensitivity of this prognostic signature were determined by the receiver operating characteristic (ROC) curve constructed with the “SurvivalROC” package. In addition, we identified copy-number alterations and performed mutation analysis of the risk genes in sarcomas using the cBioportal database (http://www.cbioportal.org/). Additionally, 53 osteosarcoma patient samples from the GSE21257 dataset were used to verify the reliability of the prognostic model.

### Independent Prognostic Analysis and Clinical Correlation Analysis

We extracted clinical information (gender, age, and metastasis status) of patients in the TARGET cohort. We implemented the “survival” package to conduct both univariate and multivariate Cox regression analysis to assess the independence of the PRS-score from other clinical variables. The R “RMS” package was then used to generate nomograms to predict survival in patients with osteosarcoma over the course of 1, 3, and 5 years. Additionally, osteosarcoma patients were divided into two subgroups according to age (≤ 18 or > 18 years old), gender (female or male), and metastasis status (M0 and M1). The R “Beeswarm,” “limma,” and “pheatmap” package was used to assess the correlation between the PRGs involved in the prognostic signature and clinical parameters mentioned above.

### Functional Enrichment Analyses

We applied the “limma” R package to identify differentially expressed genes (DEGs) in the PRS-score-classified subgroups, with a false discovery rate (FDR) < 0.05 and absolute value of the log2 fold change (|log2FC|) ≥ 1 as a threshold. We implemented the “clusterProfiler” package to conduct the Gene Ontology (GO) and Kyoto Encyclopedia of Genes and Genomes (KEGG) analysis based on the DEGs between different PRS-score subgroups, with an adjusted *p*-value (adj. P) < 0.05. Subsequently, the “GSVA” package was used to conduct the single sample Gene Set Enrichment Analysis (ssGSEA) to calculate the enrichment scores of immunological cells and functions.

### Analysis of the Immune Microenvironment Features and Immune Response

Immunoscore and stromal scores for each osteosarcoma patient were obtained using the “estimate” and “limma” packages and were used to derive tumor purity. Using the “ggpubr” and “limma” packages, we assessed the differential expression of immune checkpoints (CTLA4, PDL1, LAG3, TIGIT, TIM3, PDCD1, IDO1, and TDO2) between subgroups to estimate the predictive power of the signature for immunotherapy response.

### Statistical Analysis

We executed all statistical analyses with R software (v4.0.5). The threshold for statistical significance was taken as *p* < 0.05 if it was not explicitly stated.

## Results

### DEPRGs in Human Osteosarcoma and Normal Tissues

**FIGURE 2:**
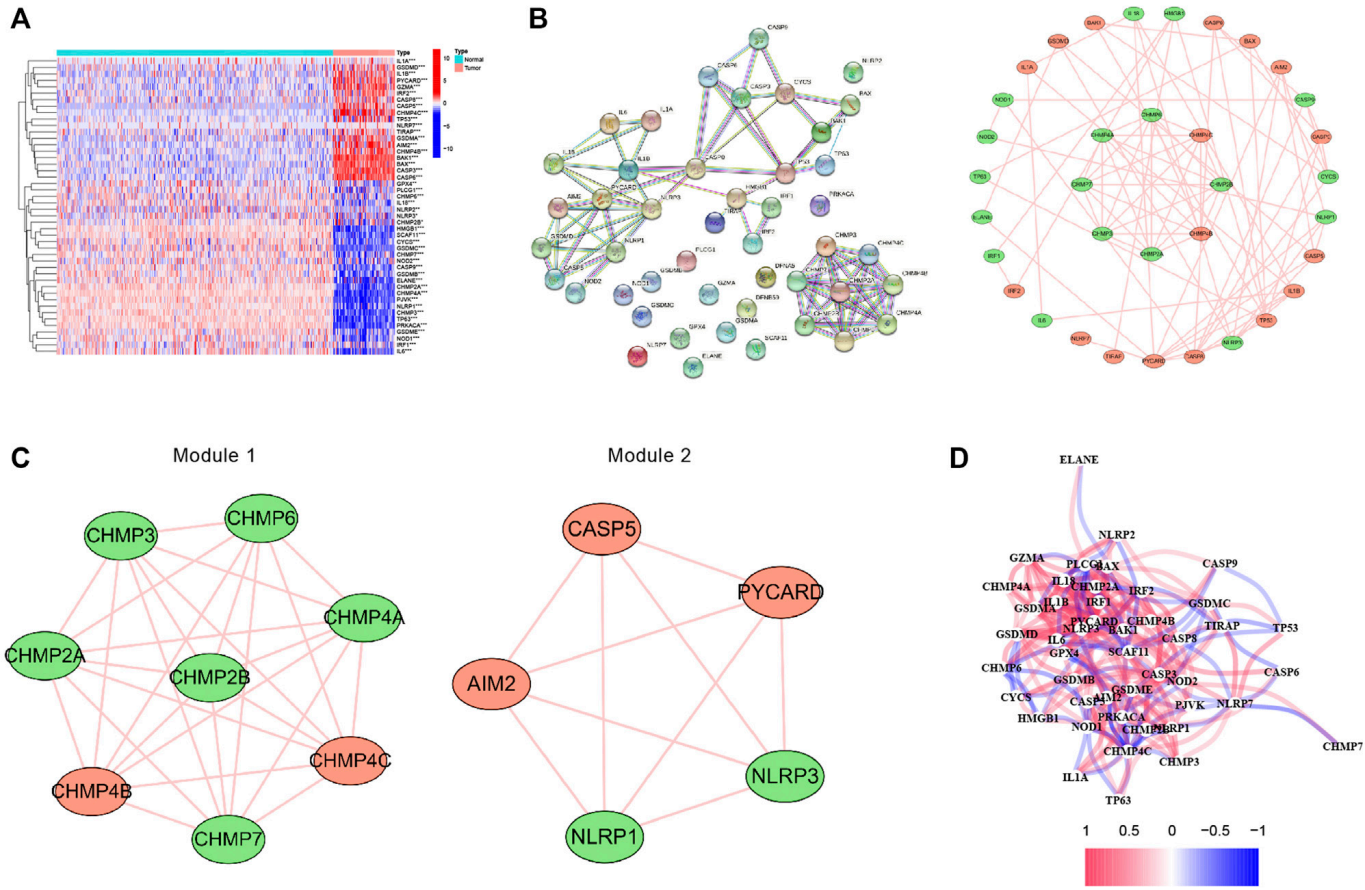
Expression and interconnectedness of the pyroptosis-related genes in osteosarcoma. **(A)** The heatmap showed the differential expressed PRGs between human osteosarcoma samples and normal muscle tissues (red: high expression level, blue: low expression level). **(B)** PPI network of differentially expressed PRGs (The red nodes indicate upregulated PRGs and the green nodes indicate downregulated PRGs) **(C)** Critical modules from the PPI network. **(D)** The correlation network of the differential expressed PRGs (red lines indicate positive correlation and blue lines indicate negative correlation). PRGs, pyroptosis-related genes.

The expression levels of 52 PRGs were compared in the human osteosarcoma samples and normal muscle tissues, and we detected 19 DEPRGs that were upregulated and 27 DEPRGs that were down-regulated using our threshold criteria (*p* value < 0.05) (Figure 2A). The PPI network of DEPRGs created with the minimum required interaction score > 0.9 is presented in Figure 2B. We then screened out the two most crucial network modules using MCODE (Figure 2C) and drew the correlation network of the differentially expressed PRGs (Figure 2D).

### Identification of Subgroups Based on PRGs by Consensus Clustering

**FIGURE 3:**
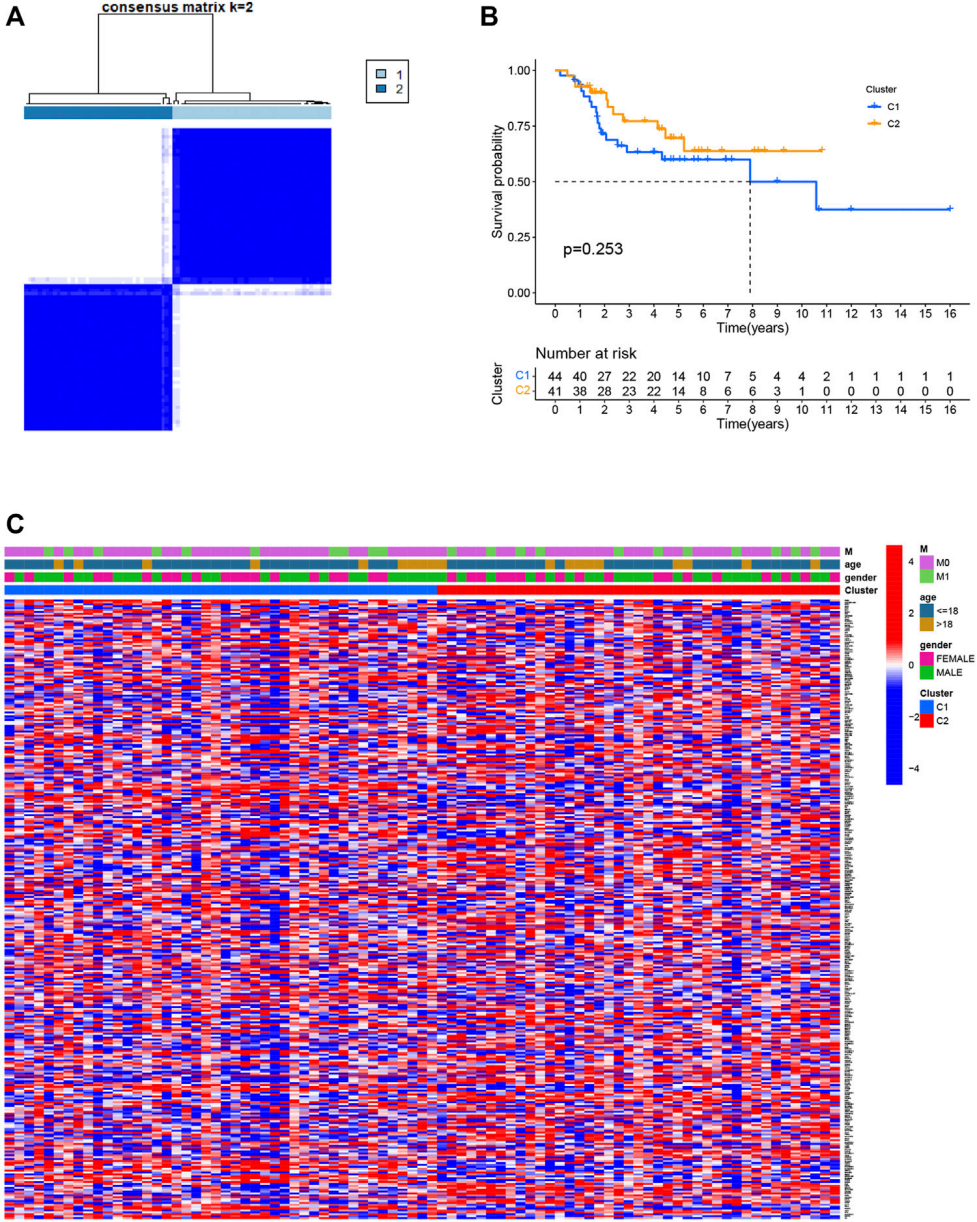
Classification of osteosarcoma patients based on pyroptosis-related regulators. **(A)** Consensus clustering of osteosarcoma patients for k = 2. **(B)** The prognostic analysis between the two pyroptosis-related clusters. **(C)** Heatmap of the differentially expressed genes and clinical characteristics between the two pyroptosis-related clusters.

Consensus clustering was used to separate all 85 osteosarcoma patients into subgroups according to the expression of PRGs. By increasing the clustering index “k” from 2 to 10, we found that k = 2 seems to be the optimal point to identify the smallest interferences and the most significant differences between clusters (Figure 3A). Consequently, patients with osteosarcoma in the training group were classified into two clusters. However, a comparison of overall survival between the two clusters revealed no significant difference (*p* = 0.253, Figure 3B). We also plotted a heatmap to express the differences in gene expression and clinical characteristics, including age (≤ 18 or > 18 years old), gender (male or female), and metastasis status (metastatic, non-metastatic) between the clusters, but we found there are little differences (Figure 3C).

### Construction of the PRG-Based Prognostic Signature

**FIGURE 4:**
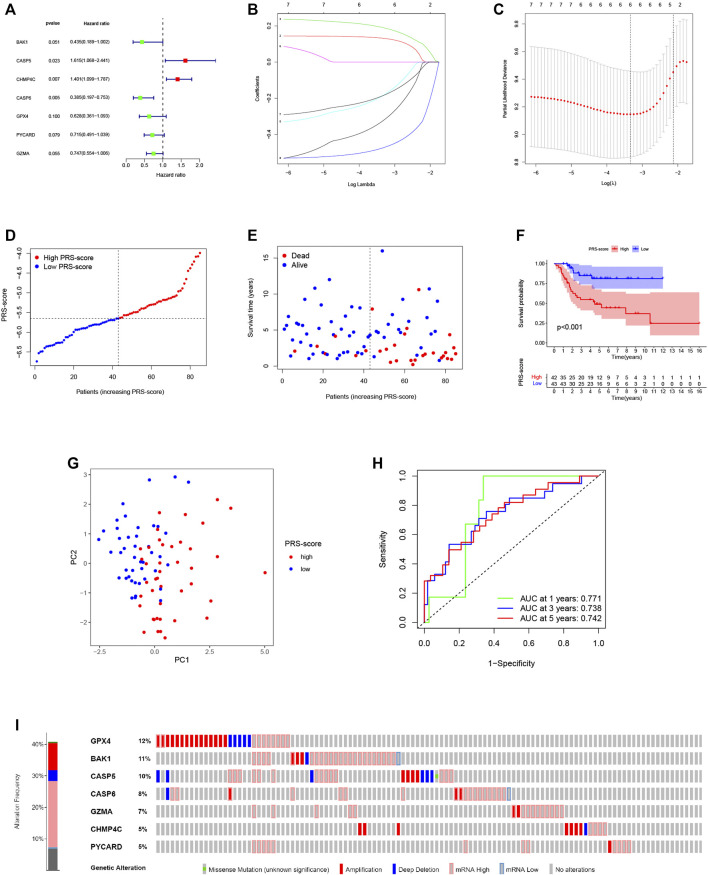
Construction of the pyroptosis-related prognostic signature for osteosarcoma. **(A,B)** Cox regression analysis of pyroptosis-related genes. **(A)** Univariate Cox regression analysis. **(B)** LASSO Cox regression analysis. **(C)** Selection of the optimal penalty parameter for LASSO regression. **(D)** The PRS-score distribution of the patients with osteosarcoma in the TARGET cohort **(E)** The survival status and survival time distribution of the patients with osteosarcoma in the TARGET cohort. **(F)** Kaplan–Meier curves of the high and low PRS-score subgroups in the TARGET cohort. **(G)** PCA plot based on the PRS-scores in the TARGET cohort. **(H)** Time-dependent ROC curve for predicting the 1-, 3-, and 5-year overall survival in the TARGET cohort. **(I)** Genomic alterations of hub genes.

To construct a pyroptosis-related prognostic model, we further screened seven-candidate prognostic PRGs by univariate Cox regression analysis (Figure 4A). Of the seven prognostic PRGs, CASP5 and CHMP4C were regarded as high-risk genes based on their HRs, whereas BAK1, CASP6, GPX4, PYCARD, and GZMA were regarded as low-risk genes. Subsequently, LASSO Cox regression analysis was performed to construct a 6-gene signature according to the optimum penalty parameter (*λ*) value (Figures 4B,C). We then divided the patients in the TARGET cohort into high and low scoring subgroups based on a composite signature score termed the “PRS-score” (PRS-score = [BAK1 expression × (−0.325)] + [CASP5 expression × (0.132)] + [CHMP4C expression × (0.191)] + [CASP6 expression × (−0.475)] + [GPX4 expression × (−0.185)] + [GZMA expression × (−0.185)]). The PRS-scores, survival status, and survival time in the two groups of patients are shown in Figures 4D,E. The results showed that patients with higher PRS-scores had worse prognoses than patients with lower PRS-scores. Kaplan-Meier curves showed that the patients in the high PRS-score group had worse OS than the patients in the low PRS-score group (*p* < 0.001; Figure 4F). Analyses of PCA revealed that high and low PRS-score patients were separated into two clusters (Figure 4G). To assess the accuracy of the signature, we then constructed a time-dependent ROC curve. We found the area under the ROC curve (AUC) was 0.771 for 1-year OS, 0.738 for 3-year OS, and 0.742 for 5-year OS, providing evidence that this six-gene prognostic model performed well as a predictor of OS (Figure 4H). Mutations and copy number alterations of the six hub genes (BAK1, CASP6, GPX4, PYCARD, GZMA, CASP5, and CHMP4C) were analyzed together using the cBioportal database. These six hub genes were altered in 99 of 241 samples (41%) (Figure 4I). Since the frequency of mutations in GPX4 and BAK1 exceeded 10%, we hypothesized that these two genes might be key therapeutic targets (Figure 4I).

### Validation of the PRG-Based Prognostic Signature

**FIGURE 5:**
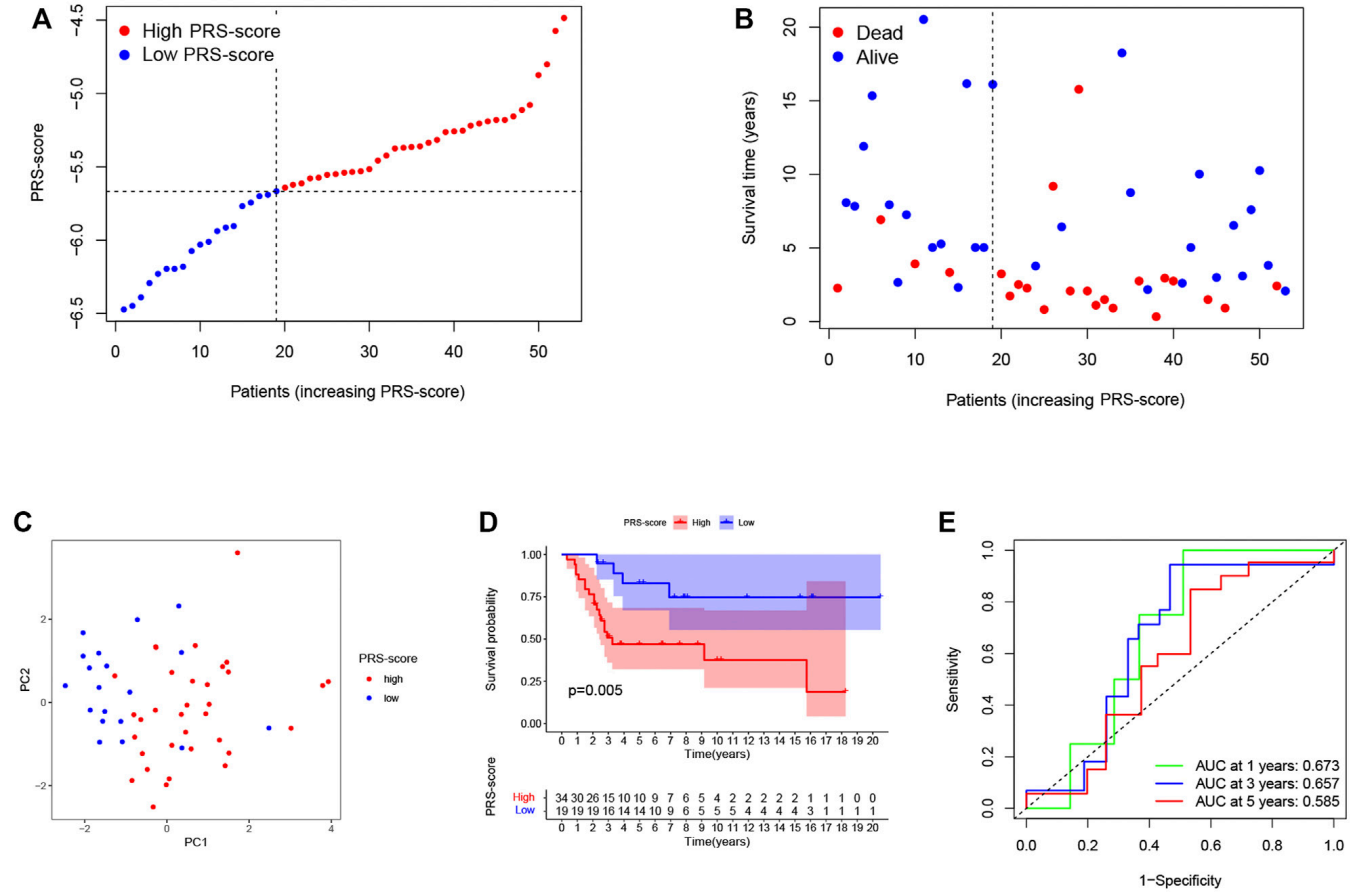
Validation of the prognostic signature in the GEO cohort. **(A)** The PRS-score distribution of the patients with osteosarcoma in the GEO cohort. **(B)** The survival status and survival time distribution of the patients with osteosarcoma in the GEO cohort. **(C)** PCA plot based on the PRS-scores in the GEO cohort. **(D)** Kaplan–Meier curves of the high and low PRS-score subgroups in the GEO cohort. **(E)** Time-dependent ROC curve for predicting 1-, 3-, and 5-year overall survival in the GEO cohort.

To reliability of this six-gene prognostic signature, a total of 53 patients from GSE21257 were used as the test set. Based on the median cut-off of the PRS-score in the TARGET cohort, patients with osteosarcoma in the GEO cohort were separated into high (n = 34) and low (n = 19) scoring groups (Figure 5A). The survival time and survival status distribution showed that patients in the low PRS-score subgroup had a higher possibility of surviving (Figure 5B). The PCA of the two subgroups showed a clear separation (Figure 5C). Furthermore, Kaplan-Meier analysis revealed that osteosarcoma patients with high PRS-scores had a significantly poorer prognosis than those with low PRS-scores (Figure 5D), with AUC = 0.673, 0.657, and 0.585 for 1, 3, and 5 years survival, respectively (Figure 5E).

### Independent Prognostic Value and Clinical Utility of the Prognostic Signature

**FIGURE 6:**
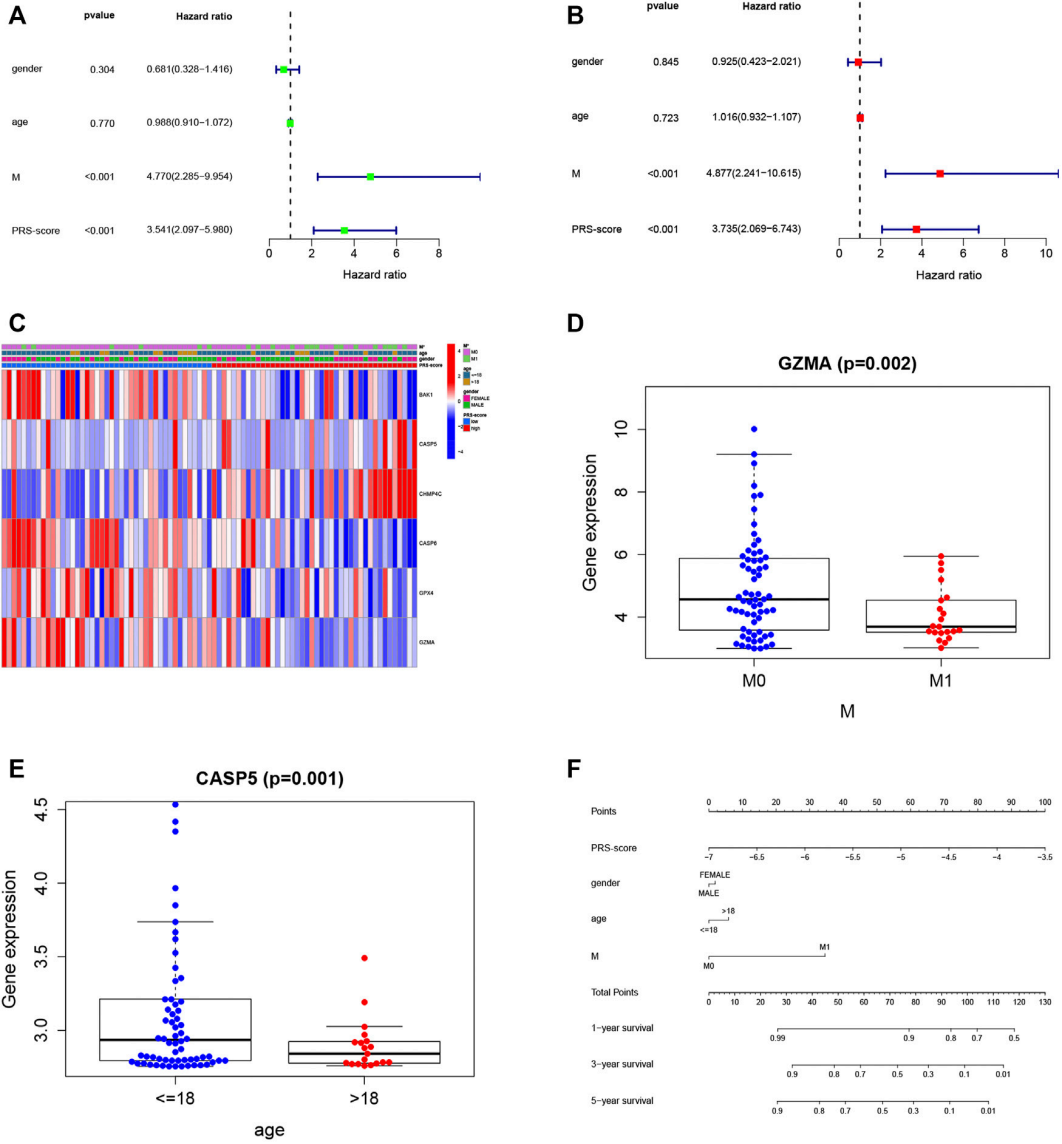
Independent prognosis analysis and clinical utility. **(A,B)** Cox regression analysis of pyroptosis-related genes **(A)** Univariate Cox regression analysis **(B)** Multivariate Cox regression analysis **(C)** Heatmap (blue: low expression level; red: high expression level) of the correlation between clinical features and the risk groups (*p < 0.05) **(D)** Relationship between GZMA and metastasis. **(E)** Relationship between CASP5 and age category. **(F)** A prognostic nomogram based on the PRG-related model for prediction of 1-, 3-, and 5-year survival rates.

We then utilized univariate and multivariable Cox regression analyses to evaluate the independent prognostic value of the model with other clinical features. The univariate Cox analysis indicated that the PRS-score (HR = 3.541, 95% CI = 2.097–5.980, *p* < 0.001) and M-stage (HR = 4.770, 95% CI = 2.285–9.954, *p* < 0.001) were significantly associated with OS (Figure 6A). The multivariate Cox analysis confirmed that the PRS-score (HR = 3.735, 95% CI = 2.069–6.743, *p* < 0.001) and M-stage (HR = 4.877, 95% CI = 2.241–10.615, *p* < 0.001) were independent factors affecting the prognosis of osteosarcoma patients (Figure 6B). We then plotted a clinical information-related heatmap for the TARGET cohort and found significant differences in M-stage distribution between low- and high-scoring subgroups (Figure 6C). The results of clinical correlation analysis showed that the M stage of osteosarcoma patients decreased with increasing GZMA expression (Figure 6D, *p* < 0.01), while osteosarcoma patients with high CASP5 expression were younger (Figure 6E, *p* < 0.01), and all results are shown in Table 1. Additionally, a pyroptosis-related signature-based nomogram showed that the OS of patients at 1, 3, and 5 years decreased with increasing PRS-score (Figure 6F).

**TABLE 1:** The relationship between PRS-scores and clinical characteristics.

Id	Gender (female, male) t (p)	Age (≤ 18, > 18) t (p)	M stage (M0, M1) t (p)
BAK1	−0.182 (0.856)	1.324 (0.197)	0.091 (0.928)
CASP5	1.257 (0.213)	**3.287(0.001)**	−0.08 (0.936)
CHMP4C	0.84 (0.404)	1.609 (0.115)	−0.568 (0.574)
CASP6	−0.724 (0.472)	0.128 (0.899)	1.965 (0.056)
GPX4	0.062 (0.951)	0.32 (0.751)	1.664 (0.107)
GZMA	0.341 (0.734)	1.434 (0.160)	**3.293(0.002)**
PRS-scores	0.742 (0.461)	−0.093 (0.926)	−**2.58(0.015)**

t, t value from Student’s t test; p: *p*-value from Student’s t test. Bold indicates statistical significance, *p* < 0.05.

### Functional Analysis of DEGs Based on PRS-Score

**FIGURE 7:**
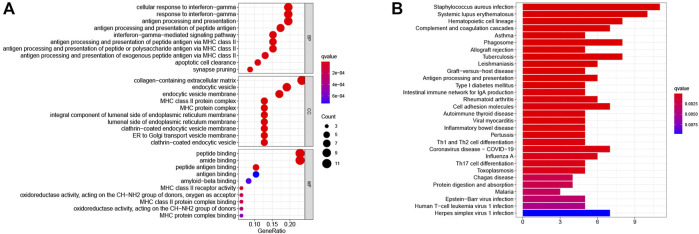
Functional enrichment analysis of DEGs between the two pyroptosis-related subgroups. **(A)** GO enrichment analysis of DEGs based on PRS-score, including BP, CC, and MF. **(B)** KEGG pathway enrichment analysis of DEGs based on PRS-score. GO, Gene Ontology; KEGG, Kyoto Encyclopedia of Genes and Genomes; BP, biological processes; CC, cell component; MF, molecular function.

To further investigate differences in PRS-score-classified subgroups, we identified 34 genes that were down-regulated and 14 genes that were up-regulated in the high PRS-score subgroup compared with the low PRS-score subgroup in the TARGET group (Supplementary Table S2). GO analysis revealed that the 48 DEGs were mainly involved in the cellular response to interferon-gamma, MHC class II protein complex, peptide binding, and amide binding (Figure 7A). According to the KEGG pathway analysis, these DEGs were primarily associated with staphylococcus aureus infection, systemic lupus erythematosus, hematopoietic cell lineage, and complement and coagulation cascades (Figure 7B).

### Analysis of Immune Microenvironment Characteristics Between Subgroups

**FIGURE 8:**
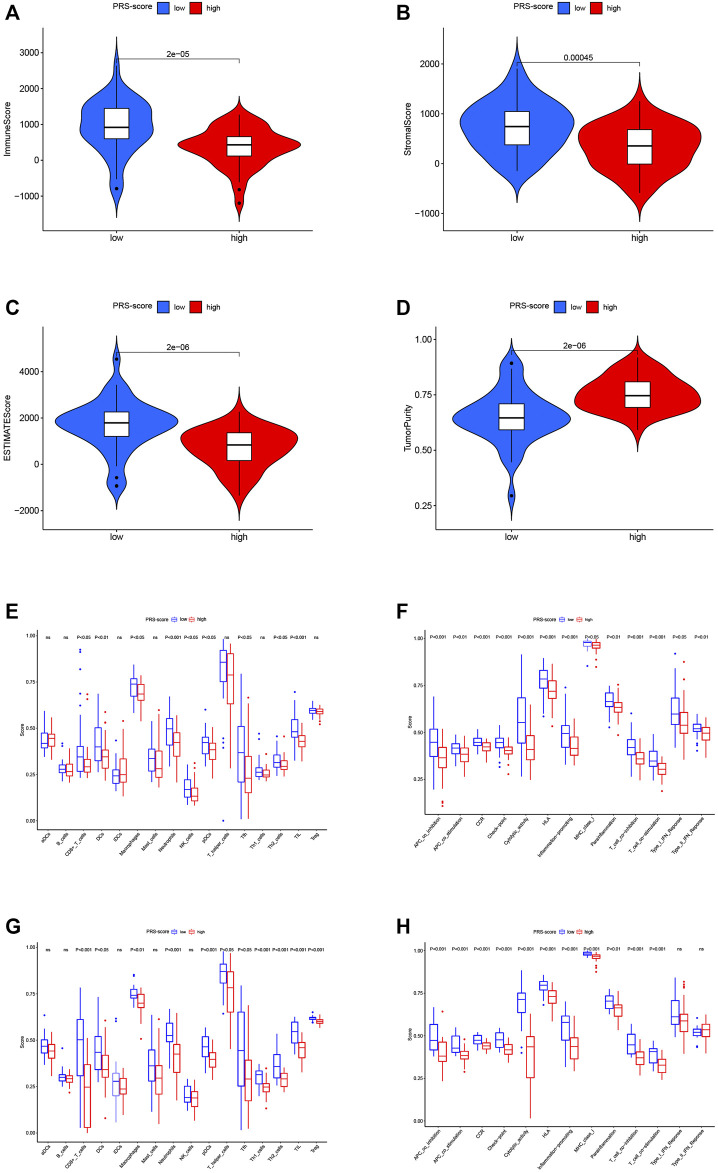
Immune characteristics analysis of the prognostic signature. **(A)** Immune scores between high and low PRS-score groups**. (B)** Stromal scores between high and low PRS-score groups **(C)** ESTIMATE scores between high and low PRS-score groups. **(D)** Tumor purity between high and low PRS-score groups **(E)** Comparisons of the level of immune cell infiltration between high and low PRS-score groups in the TARGET cohort. **(F)** Comparisons of immune functions between high and low PRS-score groups in the TARGET cohort. **(G)** Comparisons of the level of immune cell infiltration between high and low PRS-score groups in the GEO cohort. **(H)** Comparisons of immune functions between high and low PRS-score groups in the GEO cohort.

Several studies have shown that the tumor immune microenvironment correlates strongly with malignant behavior; thus, we investigated the unique features of the tumor microenvironment (TME) to distinguish between the two subgroups of patients. Based on the ESTIMATE algorithm, the overall level of immune cell infiltration and tumor purity were examined. As shown in Figures 8A–D, the immune score, stromal score, and ESTIMATE score were significantly higher in the low scoring group than in the high scoring group, while the tumor purity was lower. We then explored the distribution patterns of infiltrating immune cells in different subgroups using the ssGSEA algorithm. In the TARGET cohort, the patients in the high PRS-score group had lower levels of tumor infiltration by CD8^+^ T cells, dendritic cells (DCs), macrophages, neutrophils, natural killer cells, plasmacytoid dendritic cells (pDCs), Th2 cells, Tfh cells, and tumor-infiltrating lymphocytes (TILs) compared with the patients in the low PRS-score group (Figure 8E). All 13 immune functions were down-regulated in the patients in the high PRS-score group in comparison with the patients in the low PRS-score group (Figure 8F). In the GEO cohort, compared with the patients in the low PRS-score group, the patients in the high PRS-score group had lower levels of tumor infiltration by immune cells, including CD8^+^ T cells, DCs, macrophages, neutrophils, pDCs, TILs, T regulatory, Tfh, Th1, and Th2 cells (Figure 8G). Moreover, in contrast to the type-1 and type-2 interferon response pathways, the other 11 immune pathways had lower activity in the high PRS-score group than in the low PRS-score group (Figure 8H). Our investigation showed that PRS-scores were associated with immune characteristics and that elevated immune activity in the low-scoring samples may contribute to the antitumor effect in osteosarcoma.

**FIGURE 9:**
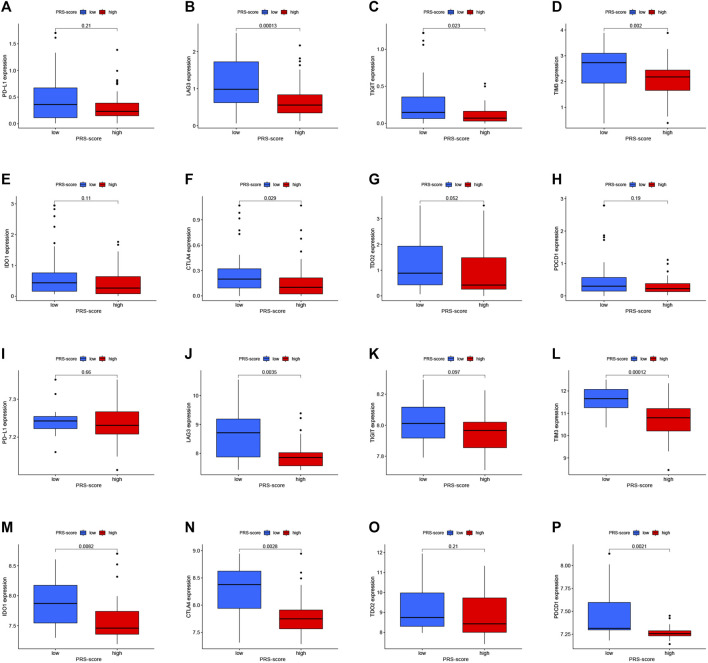
Immune checkpoint molecules expression analysis. **(A–H)** The expression levels of Immune checkpoint molecules, including PD-L1 **(A)**, LAG-3 **(B)**, TIGIT **(C)**, TIM-3 **(D)**, IDO1 **(E)**, CTLA-4 **(F)**, TDO2 **(G)**, and PDCD1 **(H)** between high and low PRS-score groups in the TARGET cohort. **(**I**–**P**)** The expression levels of Immune checkpoint molecules, including PD-L1 **(I)**, LAG-3 **(J)**, TIGIT **(K)**, TIM-3 **(L)**, IDO1 **(M)**, CTLA-4 **(N)**, TDO2 **(O)**, and PDCD1 **(P)** between high and low PRS-score groups in the GEO cohort.

In addition, we analyzed the changes in immune checkpoint expression between the high and low PRS-score groups. Figures 9A–H shows that in the TARGET cohort, LAG3 (*p* = 1.3e-04), TIGIT (*p* = 0.023), TIM3 (*p* = 0.002), and CTLA4 (*p* = 0.029) expressions were down-regulated in the high-scoring group in comparison to the low-scoring group. On the other hand, as the PRS-score increased, the expression of LAG3 (*p* = 0.0035), TIM3 (*p* = 1.2e-04), IDO1 (*p* = 0.0082), CTLA4 (*p* = 0.0028), and PDCD1 (*p* = 0.0021) in patients with osteosarcoma also decreased in the GEO cohort (Figures 9I–P).

## Discussion

Pyroptosis, a form of programmed cell death, was found to play a dual role in both promoting and inhibiting the growth of different tumor cells (Loveless et al., 2021). Several recent studies have highlighted the relevance of pyroptosis-related genes as candidate biomarkers for prognosis and therapeutic response in patients with different cancer types (Ju A. et al., 2021; Lin W. et al., 2021; Shao et al., 2021; Ye et al., 2021). In the current study, we identified the mRNA levels of 52 pyroptosis-related genes in osteosarcoma and normal tissues based on public databases and found that most of these genes were differentially expressed. However, DEPRGs-based consensus clustering analysis produced two clusters that showed no significant differences in clinical characteristics. Subsequently, we performed univariate and LASSO Cox regression analyses to further identify six prognosis-related RPGs. To further explore their biological function and clinical significance, we also performed survival and ROC analyses to develop an accurate pyroptosis-related prognostic signature in osteosarcoma. Subsequently, ssGSEA found that the high-scoring group had lower levels of immune infiltration and fewer immune-related pathways than the low-scoring group. These results suggest that the novel pyroptosis-related genes signature has the potential to predict prognosis accurately and could provide new diagnostic biomarkers and therapeutic targets for patients with osteosarcoma.

As a result of the present study, we constructed a 6-gene pyroptosis-related signature, including BAK1, CASP5, CASP6, GPX4, GZMA, and CHMP4C. Notably, six genes involved in this signature have been implicated in apoptotic pathways as well (Li et al., 2015; Skotte et al., 2017; Wu et al., 2019; Zhou et al., 2020; Darweesh et al., 2021; Ding et al., 2021). Following apoptotic signals, caspase 8 and caspase 3 initiate pyroptosis by processing GSDMC and GSDME, respectively (Liu et al., 2021). The close relationship between pyroptosis and apoptosis may explain the dual role of these genes. Caspase 5 is an essential player in canonical or noncanonical inflammasome-induced pyroptosis. Upon activation, caspase-5 can act on the GSDMD, leading to the formation of cell membrane pores. Activated caspase-5 can also interact with caspase-1 to promote its activation, and the latter cleaves the precursors of IL-1+ and IL-18 to form active IL-1+ and IL-18, which are released through the channels formed by GSDMD-cNT and lead to pyroptosis (Kayagaki et al., 2015; Xia et al., 2019). Studies have reported that caspase-5 is associated with various malignancies, including gastric cancer, cervical cancer, lung cancer, and human glioblastoma (Babas et al., 2010; Zhou et al., 2018; Wang et al., 2019). Caspase-6 plays a vital role in promoting cell death, ZBP1-mediated inflammasome activation, and host defense during IAV infection (Zheng et al., 2020; Zheng and Kanneganti, 2020). In addition, caspase-6 can also be involved in cancer progression by regulating tumor apoptosis and metastasis (Capo-Chichi et al., 2018). GPX4 was found to negatively regulate Gasdermin D-mediated pyroptosis in lethal polymicrobial sepsis by reducing lipid peroxidation; in contrast, conditional GPX4 knockdown in myeloid cells triggers macrophage pyroptosis with caspase-1/caspase-11-GSDMD-phospholipase C gamma 1 axis. (Kang et al., 2018). Chen et al. (2021a) found that circKIF4A promoted papillary thyroid tumors by sponging miR-1231 and upregulating GPX4 expression. GPX4 is also a ferroptosis-related factor playing an essential role in iron-dependent oxidative cell death driven by lipid peroxidation (Chen X. et al., 2021). Lin H. et al. (2021) discovered that upregulation of HMOX1 to inhibit GPX4 expression induced ferroptosis in osteosarcoma cells by increasing reactive oxygen species levels, malondialdehyde levels, and intracellular ferric ion level. GZMA from cytotoxic lymphocytes enhances antitumor immunity and promotes tumor clearance by cleavage of GSDMB triggering pyroptosis (Zhou et al., 2020). On the other hand, GZMA acts as a pro-inflammatory cytokine to promote cancer development (van Daalen et al., 2020); for instance, GZMA deficiency inhibits colon cancer development and inflammatory response in colon tissue through the NF-κB-IL-6-pSTAT3 axis (Santiago et al., 2020). The polymorphism of CHMP4C increased the cancer susceptibility and was imbalanced in many cancers, including lung, ovarian, prostate, and cervical cancers (Lin S. L. et al., 2020). Another study showed that CHMP4C is also an autophagy-related gene, and its participation in the construction of risk models could effectively predict the prognosis of cervical cancer patients and help develop precise treatment strategies (Shi et al., 2020). Notably, similar to CHMP4C, BAK1 was found to be an apoptosis and pyroptosis-related gene (Cowan et al., 2020; Deo et al., 2020). BAK1 is a member of the Bcl-2 family and can induce mitochondria-mediated apoptosis via regulating the release of cytochrome c (Vervliet et al., 2016). Recent studies have shown that miR-125b, miR-410, and miR-103a-3p could all directly target BAK1 to inhibit apoptosis, and upregulation of BAK1 may contribute to the treatment of cisplatin-resistant non-small cell lung cancer (Wen et al., 2018; Wang H. et al., 2021; Zhang et al., 2021). A prognostic signature based on 14 genes, including BAK1, was able to predict the survival outcome for patients with osteosarcoma (Qi et al., 2021). We also found that PYCARD, although not included in the construction of the model, was also associated with patient outcomes. PYCARD is an adaptor protein that helps form inflammasomes, which contribute to inflammation by promoting the release of the active IL-1β and IL-18 (Hoffman and Wanderer, 2010; Protti and De Monte, 2020). Inflammation is commonly thought to contribute to driving tumor growth, metastasis, and immune escape; for example, IL-1 promotes tumor angiogenesis, recruitment of myeloid cells and contributes to tumor metastasis by recognizing endothelial cell adhesion molecules (Mantovani et al., 2018; Karin and Shalapour, 2021). On the other hand, PYCARD was found to be silenced by promoter methylation in various cancer cells, suggesting its anti-tumor role as a pro-apoptotic factor (Agrawal and Jha, 2020). These studies further confirmed the potential prognostic value of the identified pyroptosis-related genes in osteosarcoma. However, the exact mechanism of their involvement in pyroptosis in osteosarcoma needs to be verified by further *in vivo* and *in vitro* experiments.

The enrichment analysis results showed that the DEGs between high and low PRS-score subgroups were mainly enriched in interferon-gamma mediated signaling pathways, antigen processing, and peptide antigens presented via MHC class II, peptide binding. The MHC-II is the critical component of adaptive anti-tumor immunity, and its upregulation is closely associated with increased levels of interferon-gamma in tumors (Dubrot et al., 2014; Cook et al., 2021). During inflammation, epithelial cells could act as accessory antigen-presenting cells along with the expression of MHC-II (Ghasemi et al., 2020). Tumor-specific MHC-II expression is associated with better prognosis, T-cell infiltration, higher levels of Th1 cytokines, and sensitivity to anti-PD-1 therapies (Johnson et al., 2020). Lu et al. (2017), used the adoptive transfer of MHC-II-restricted tumor-reactive T cells in patients with metastatic cancer (which contained patients with osteosarcoma) and achieved different degrees of tumor regressions in these patients. Coincidentally, the ssGSEA results indicated lower levels of principal anti-tumor infiltrating immune cells in the high PRS-score group, providing further evidence that these genes may play a role in anti-tumor immunity. Studies have shown that chimeric antigen receptor T-cell immunotherapy, a potent option for drug-resistant tumors, has transformed the treatment of drug-resistant hematologic malignancies yet remains largely ineffective against solid tumors, which may be related to the tumor immune microenvironment, the stromal barrier, and the lack of surface tumor-specific targets (Titov et al., 2021). Therefore, we used the ESTIMATE algorithm to examine the distribution of immune scores, stromal scores, and tumor purity in osteosarcoma patients in high and low PRS-score groups. We found that the low-scoring group showed higher immune scores, ESTIMATE scores. Consistent with these results, the high-scoring group had high tumor purity. The PRS-score may help assess the immune microenvironment features of patients and thus predict their sensitivity to immunotherapy, which will help to guide individualized anti-tumor treatment strategies. Finally, we evaluated the differences in immune checkpoint expression between the two subgroups to determine whether patients would benefit from immune checkpoint inhibitor therapy.

In previous studies, several prognostic signatures have been constructed from different perspectives to predict the prognosis of oeosarcoma. Jiang et al. (2021) created a hypoxia gene-based signature to predict the survival in childhood osteosarcoma. Wang et al. developed a new classification system of osteosarcoma based on immune features and identified TYROBP as a key immune regulatory gene (Wang X. et al., 2021). Qi et al. identified a prognostic signature of osteosarcoma based on 14 autophagy-related genes that can guide clinical decisions in treating osteosarcoma (Qi et al., 2021). Nonetheless, no research has so far concentrated on PRGs-related models, and the current study was designed to fill the vacancy in PRGs-based models for predicting outcomes. Of course, there are inevitably some limitations to this study. Firstly, the verification cohort has a relatively small sample size due to the inherent property of osteosarcoma. Secondly, it lacks experimental work, and the molecular mechanisms of its specific involvement still need further study.

In conclusion, we have developed a novel prognostic model based on six pyroptosis-related genes through comprehensive and systematic bioinformatics analysis, providing an essential foundation for future studies of the association between pyroptosis-related genes and immunity in osteosarcoma.

## Data Availability

The original contributions presented in the study are included in the article/Supplementary Material, further inquiries can be directed to the corresponding authors.
